# Factors Associated with Medication Non-Adherence among Patients with Multimorbidity and Polypharmacy Admitted to an Intermediate Care Center

**DOI:** 10.3390/ijerph18189606

**Published:** 2021-09-12

**Authors:** Javier González-Bueno, Daniel Sevilla-Sánchez, Emma Puigoriol-Juvanteny, Núria Molist-Brunet, Carles Codina-Jané, Joan Espaulella-Panicot

**Affiliations:** 1 Pharmacy Department, Consorci Hospitalari de Vic, 08500 Barcelona, Spain; ccodinajane@gmail.com; 2 Central Catalonia Chronicity Research Group (C3RG), University of Vic—Central University of Catalonia (UVIC-UCC), 08500 Barcelona, Spain; danielsevillasanchez@gmail.com (D.S.-S.); nmolist@hsc.chv.cat (N.M.-B.); jespaulella@hsc.chv.cat (J.E.-P.); 3 Pharmacy Department, Parc Sanitari Pere Virgili, 08023 Barcelona, Spain; 4 Epidemiology Unit, Consorci Hospitalari de Vic, 08500 Barcelona, Spain; epuigoriol@chv.cat; 5 Tissue Repair and Regeneration Laboratory (TR2Lab) Group, Faculty of Sciences and Technology & Faculty of Medicine, University of Vic—Central University of Catalonia (UVIC-UCC), 08500 Barcelona, Spain; 6 Department of Geriatrics, Consorci Hospitalari de Vic & Fundació Hospital de la Santa Creu de Vic, 08500 Barcelona, Spain

**Keywords:** multimorbidity, elderly, polypharmacy, inappropriate prescribing, medication adherence

## Abstract

Identifying determinants of medication non-adherence in patients with multimorbidity would provide a step forward in developing patient-centered strategies to optimize their care. Medication appropriateness has been proposed to play a major role in medication non-adherence, reinforcing the importance of interdisciplinary medication review. This study examines factors associated with medication non-adherence among older patients with multimorbidity and polypharmacy. A cross-sectional study of non-institutionalized patients aged ≥65 years with ≥2 chronic conditions and ≥5 long-term medications admitted to an intermediate care center was performed. Ninety-three patients were included (mean age 83.0 ± 6.1 years). The prevalence of non-adherence based on patients’ multiple discretized proportion of days covered was 79.6% (*n* = 74). According to multivariable analyses, individuals with a suboptimal self-report adherence (by using the Spanish-version Adherence to Refills and Medications Scale) were more likely to be non-adherent to medications (OR = 8.99, 95% CI 2.80–28.84, *p* < 0.001). Having ≥3 potentially inappropriate prescribing (OR = 3.90, 95% CI 0.95–15.99, *p* = 0.059) was barely below the level of significance. These two factors seem to capture most of the non-adherence determinants identified in bivariate analyses, including medication burden, medication appropriateness and patients’ experiences related to medication management. Thus, the relationship between patients’ self-reported adherence and medication appropriateness provides a basis to implement targeted strategies to improve effective prescribing in patients with multimorbidity.

## 1. Introduction

As the global population is ageing, the prevalence of people living with multimorbidity, defined as the presence of two or more chronic conditions, becomes increasingly common [1]. Elderly individuals with multimorbidity are associated with poorer health outcomes, including lower health-related quality of life, higher utilization of health care services, increased disability, frailty and mortality [2].

Polypharmacy, i.e., the use of five or more medications, is steadily rising in older adults due to the strict application of clinical practice guidelines focused on patients with single chronic conditions [3]. Medication non-adherence is a frequent consequence of polypharmacy. Among medication non-adherence negative effects are increased morbidity, mortality and costs. Patients with multimorbidity are more likely to have polypharmacy and frailty, making them especially vulnerable to non-adherence and the associated consequences [4,5].

Adherence to medications is the process by which patients take their medication as prescribed, further divided into three quantifiable phases: initiation, implementation and discontinuation. In accordance with ABC taxonomy, non-adherence can occur due to a late or non-initiation of the prescribed treatment, suboptimal implementation of the dosing regimen or early discontinuation [6]. Medication non-adherence is a multi-factorial process caused by a highly complex interplay between many modifiable and unmodifiable determinants, which can be categorized into five dimensions (socioeconomic, patient-related, therapy-related, condition-related and health system-related) [7]. The more complex a treatment regimen, the higher the risk of non-adherence. Medication adherence also changes due to adverse drug events (ADEs) or patients’ inadequate knowledge and/or beliefs about drug therapy [8]. However, most of the research on medication non-adherence determinants has focused on patients with single chronic conditions despite the urgent need to understand what influences patients with multimorbidity to take their medicines [9].

There is no standard criterion available to measure adherence in patients receiving polypharmacy, so appropriate measurement of multiple medication adherence remains a challenge [10]. Self-report methods are the most frequently used indirect methods for measuring medication adherence [11]. Nevertheless, the use of self-report measures in monitoring medication adherence remains controversial as they can be biased by a ceiling effect [12]. In contrast, self-report measures might help inform tailored interventions by identifying individual barriers and beliefs that are influencing medication adherence [13]. Furthermore, the use of dispensing data has been a staple in adherence measurements due to their validity, relative accessibility and inexpensiveness [14]. This allows the calculation of quasi-objective measures of adherence such as medication possession ratio (MPR) and proportion of days covered (PDC), based on the percentage of days the patient has medication available. Formulas for derivation of MPR or PDC differ between studies [10]. Multiple discretized PDC might be considered an estimate of choice due to its sensitivity, specificity and applicability [15].

Identifying variables influencing medication non-adherence in patients with multimorbidity by means of appropriate estimates would provide a step forward in developing patient-centered strategies to optimize their care.

The main aim of this study was to examine factors associated with the likelihood of medication non-adherence among non-institutionalized older patients with multimorbidity and polypharmacy admitted to an intermediate care center.

## 2. Materials and Methods

### 2.1. Study Site and Participants

This was a cross-sectional study representing a substudy of a quasi-experimental (before–after) research, the main goal of which was to assess the efficacy of a patient-centered prescription model to improve medication adherence and effective prescribing (defined as the process by which a provider selects the best medication regimen for accomplishing clinical and patient-centered goals after weighing shared decision making) [16] in 93 patients with multimorbidity. The study setting was a convalescent and rehabilitation ward in San Jaume de Manlleu Hospital, a 66-bed intermediate care step-down community hospital located close to Vic University Hospital, an acute care teaching hospital. Both are referral care centers for the Osona county, a mixed urban–rural district in Barcelona, Spain, with a population of 160,000 inhabitants, 3.3% aged 85 years or more.

Patients were consecutively considered for inclusion if they met the following eligibility criteria: older people (≥65 years) with ≥2 chronic conditions (from the expanded diagnostic clusters within the Johns Hopkins University Adjusted Clinical Groups (ACG) system) [17] who were receiving polypharmacy (≥5 regularly scheduled long-term (≥3 months) medications) before hospital admission. Patients were excluded from study participation if any of the following was applicable: limited life expectancy (using NECPAL CCOMS-ICO^®^ tool criteria) [18], nursing home residents or hospital admissions during the 6 months prior to inclusion in the study (to ensure an appropriate assessment of medication adherence). Potential participants were screened for eligibility before hospital discharge. In case of agreeing to participate, they were assigned a code number prior to data entry to maintain anonymity. From April 2019 to February 2020, potential participants were enrolled in the study if informed consent was provided by them or by their relatives in case of them being unable to provide consent, as approved by the ethics committee. Recruitment finished just before COVID-19 outbreak hit the Osona county.

Recommendations from STROBE guideline [19] and ESPACOMP Medication Adherence Reporting Guideline (EMERGE) [20] were followed.

### 2.2. Data Collection

#### 2.2.1. Demographic and Clinical Data

The following demographic and clinical data were collected: age, sex, chronic conditions (from the expanded diagnostic clusters within the Johns Hopkins University ACG system) [17], frailty index (Frail-VIG) [21], Barthel index for activities of daily living [22] and cognitive impairment (MMSE) [23]. Demographic and clinical data were collected from patient’s electronic medical records and by interviewing the patient and/or main caregiver. Frailty index, Barthel index and cognitive status corresponded to the patient’s status before hospitalization

#### 2.2.2. Medication-Related Data

The following medication-related data were collected:

- *Long-term medications:* Estimated as the sum of every regularly scheduled medication intended to be administered for a period ≥ 3 months.
- *Hyperpolypharmacy:* Also known as excessive polypharmacy, defined as the use of 10 or more regularly scheduled long-term medications [24].
- *Medication regimen complexity:* Assessed as a continuous variable for all long-term medications (defined as regularly scheduled long-term medications plus when required (prn) medications) on admission using the Spanish-version Medication Regimen Complexity Index (MRCI) [25]. Furthermore, regimen complexity was also categorized as low (equivalent to MRCI < 20), medium–high (MRCI 20–39.5) or excessive (MRCI ≥ 40).
- *Anticholinergic and sedative risk score:* Assessed by using the Drug Burden Index (DBI) [26,27] for every regularly scheduled long-term medication prescribed before admission.
- *Potentially inappropriate prescribing (PIP).* Every patient’s treatment plan was analyzed by a geriatrician and a clinical pharmacist through the 4-stage patient-centered prescription (PCP) model, which centers therapeutic decisions on the patient’s global assessment. Such an approach represents an advanced medication review framework [28], which has been associated with reducing inappropriate prescribing and medication burden in patients with multimorbidity [29,30,31]. The PCP model was developed by the Central Catalonia Chronicity Research Group (C3RG) and its implementation in clinical practice is recommended by the Department of Health, Government of Catalonia (Spain) for elderly and frail patients with multimorbidity [32]. PIP was considered on admission in any of the following circumstances: absence of evidence-based indication, dosing unnecessarily high considering the patient’s specific therapeutic objectives, unacceptable ADEs, contraindicated drug–drug interaction, unnecessary therapeutic duplication, inappropriate dosing or pharmaceutical dosage form or any prescription characterized as potentially inappropriate by the American Geriatrics Society 2019 Updated Beers criteria^®^ [33]. PIP was assessed as a continuous variable and categorized as moderate (≥2) and high (≥3) PIP burden.
- *Self-reported adherence.* A self-report measure of medication adherence was performed by using the Spanish-version Adherence to Refills and Medications Scale (ARMS-e) [34]. This scale consists of 12 items that assess patients’ ability to take and refill medications. Response options are on a Likert scale with responses of “none”, “some”, “most” or “all” of the time, which are given values from 1 to 4. Items were written so that a lower score is indicative of better medication adherence. The ARMS-e total score ranges from 12 to 48. Therefore, a patient that does not have any non-adherence issue will score 12, with higher scores indicating worst adherence. Written permission for conducting adherence assessments was obtained from the original developer of the English-version ARMS [35]. ARMS-e total score, based on an ordinal scale, was dichotomized through the median score using a cutoff value of 12 (optimal self-reported adherence = 12 and suboptimal self-reported adherence > 12).
- *Medication management at home:* Patients were grouped on three levels (independent, partially or totally assisted) with regard to their autonomy for medication administration and medication refills before hospital admission.
- *Multiple discretized PDC:* Medication adherence was assessed during a 6-month period before admission using the multiple discretized PDC, which was considered the main dependent variable. PDC for all regularly scheduled long-term medications was estimated as the sum of the days supplied for each medication according to electronic linked pharmacy claims data. At least two prescription refill dates during a period ≥ 90 days were required for each medication to calculate this ratio. The PDC rate was converted to a percentage based on the percentage of days covered by dispensed medication. Patients were considered adherent if PDC for each medication was ≥80% (excluding last refill) [15].

### 2.3. Statistical Analysis

Statistical analysis was performed using SPSS Version 27.0 (IBM SPSS Statistics, Armonk, NY, USA).

Results for categorical variables were expressed as absolute and relative frequencies and results for continuous variables as means and standard deviations (SD) if they followed a normal distribution, or as the median and inter-quartile range (IQR) if they did not follow a normal distribution.

Comparisons between adherent and non-adherent patients (by considering their multiple discretized PDC before admission) were performed using Student’s *t* test for parametric continuous variables (normally distributed) or the Mann–Whitney U test for nonparametric continuous variables (not normally distributed). The chi-square test (or Fisher’s exact test where appropriate) was applied to compare categorical variables between adherent and non-adherent patients.

Multivariable logistic regression analysis was conducted to assess the impact of each predictor on medication non-adherence before admission. The multivariate model was used for variables that had a *p* value < 0.10 in the bivariate analyses using stepwise regression.

Statistical significance was set at a two-sided *p* value of 0.05.

## 3. Results

**Figure 1:**
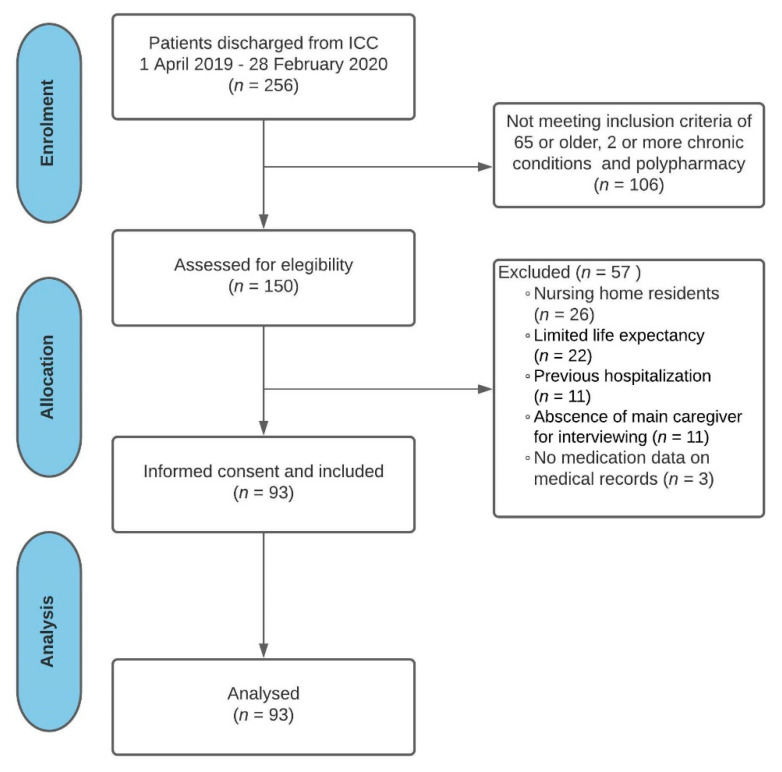
Flow diagram: study participation and selection process. Abbreviations: ICC: intermediate care center.

Out of the 256 patients who were eligible to participate in the study, a total of 93 non-institutionalized older patients with multimorbidity and polypharmacy were finally included (Figure 1). Table 1 shows demographics in addition to clinical and medication characteristics of the study participants.

The average age was 83.0 (SD 6.1) years, and the majority of patients were female (65.6%, *n* = 61). The patients had a mean number of chronic conditions of 7.4 (SD 1.8). About 80.7% (*n* = 75) had mild or moderate frailty, 53.8% (*n* = 50) had mild-to-moderate dependence for activities of daily living and 43% (*n* = 40) had cognitive impairment.

Patients were receiving an average of 8.8 (SD 2.8) regularly scheduled long-term medications, 62% (*n* = 57) of them being exposed to moderate-high or excessive medication regimen complexities. Moreover, a high prevalence of PIP was characterized with at least one PIP detected in almost every patient (98.9%, *n* = 92). Most of the study population (75.3%, *n* = 70) reported a suboptimal adherence despite a substantial proportion of them being partially or totally assisted for medication refills (62.4%, *n* = 58) or medication administration (50.5%, *n* = 47) at home.

The prevalence of non-adherence based on patients’ multiple discretized PDC was 79.6% (*n* = 74). Differences in demographic, clinical and medication characteristics between non-adherent and adherent patients in accordance with their multiple discretized PDC are shown in Table 1. There were no significant differences in demographic and clinical characteristics between both groups.

According to the bivariate analyses (Table 2), being exposed to a higher number of regularly scheduled long-term medications was positively associated with the likelihood of being non-adherent (OR = 1.46, 95% CI 1.13–1.89, *p* = 0.004). In the same manner, a 1-point increase in medication regimen complexity was associated with 7% higher odds of being non-adherent (OR = 1.07, 95% CI 1.01–1.34, *p* = 0.025). Compared with those with low medication regimen complexities, the OR for those exposed to moderate-high medication regimen complexities was 5.94 (95% CI 1.73–20.32, *p* = 0.005). Furthermore, hyperpolypharmacy was also associated with a higher probability of being non-adherent (OR = 4.06, 95% CI 1.09–15.15, *p* = 0.037).

A positive association between medication appropriateness and medication adherence was revealed through the OR for those exposed to high PIP burden (OR = 4.53, 95% CI 1.22–16.89, *p* = 0.024).

Further, a 1-unit increase in ARMS-e total score was associated with a 38% increase in patients’ odds of being non-adherent (OR = 1.38, 95% CI 1.13–1.67, *p* = 0.001). Similarly, suboptimal self-reported adherence (equivalent to ARMS > 12 points) was also significantly associated with medication non-adherence (OR = 9.82, 95% CI 3.17–30.42, *p* < 0.001).

The following variables, although not significantly associated with medication non-adherence at bivariate analyses, were included in the multivariate regression model due to a *p* value < 0.10: number of chronic conditions, number of PIP, moderate PIP and being partially assisted for medication refills at home.

Based on the multivariable logistic regression model (Table 2), individuals with a suboptimal self-reported adherence were more likely to be non-adherent to medications (OR = 8.99, 95% CI 2.80–28.84, *p* < 0.001). Having a high PIP burden (OR = 3.90, 95% CI 0.95–15.99, *p* = 0.059) was barely below the level of significance.

## 4. Discussion

By using pharmacy claims data to estimate multiple discretized PDC, this study examined factors associated with medication non-adherence in non-institutionalized older patients with multimorbidity and polypharmacy, most of them with clinical frailty, dependence for activities of daily living and regularly exposure to a high medication burden. The results demonstrated that suboptimal self-reported adherence characterized by using the Spanish-version ARMS is strongly associated with medication nonadherence based on pharmacy claims data. In addition, being exposed to a high PIP burden appears to also influence medication non-adherence. These two factors combined seem to capture most of the non-adherence determinants identified in bivariate analyses, including medication burden, medication appropriateness and patients’ experiences related to medication taking and refills.

Our results highlight the relevance of effective prescribing regarding the care of older patients with multimorbidity. Medication appropriateness and effective prescribing are both close but not interchangeable terms as the latter includes discussion of solutions to patients’ perceived barriers to obtaining and taking medications that are part of an agreed-upon treatment plan [36]. Medication adherence and medication appropriateness are therefore necessarily linked through effective prescribing. Considering our results, the use of subjective and objective measures within strategies aimed to enhance effective prescribing would lead us a systemic approach to identify patients at risk for medication non-adherence.

Our results are consistent with previous findings focused on the assessment of factors related to medication non-adherence among chronic patients. Medication burden assessed through the number of long-term medications, prevalence of hyperpolypharmacy or medication regimen complexity is a well-established determinant that negatively affects medication adherence [37,38,39]. Our findings would reinforce prior evidence through a sample of frail patients with multimorbidity.

Nevertheless, the negative association between cumulative PIP and medication adherence reported in our study has not been characterized before in patients with multimorbidity. This is of particular importance due to the high prevalence of PIP in older patients admitted to intermediate care facilities [40]. PIP has been related to higher incidence of ADEs [41,42], which could negatively affect medication adherence, thereby explaining the link between PIP and medication adherence. Moreover, medication burden has been identified as a risk factor for PIP and ADEs [43,44], thus reinforcing the usefulness of PIP as a predictor of medication non-adherence.

Furthermore, evidence regarding an association between ARMS scores and pharmacy claims data has been lacking to date in very elderly patients with frailty. The ARMS-e has been cross-culturally adapted to Spanish, but a formal validation is still not available [34]. In the meantime, self-report adherence measures using the ARMS-e seem to reflect medication adherence estimated through a quasi-objective measure such as the multiple discretized PDC. Previous association suggests the usefulness of the ARMS-e for a qualitative screening of non-adherent elderly patients with multimorbidity.

Since ARMS allows the assessment of patients’ experiences related to medication taking and refills, our results would be consistent with those of Kvarnström et al., pointing out communication and information on medicines among the critical factors for medication adherence [45]. This is aligned with growing consensus about including patients’ experiences and preferences within advanced medication review frameworks to overcome medication adherence barriers and align medication therapies with patients’ goals of care [46].

To the best of our knowledge, this is the first study assessing factors associated with medication non-adherence by using the multiple discretized PDC, which has shown to double specificity as compared to other quasi-objective adherence measures such as the mean PDC or MPR [15]. This fact might justify the low adherence rates described in our study in comparison with previous research [47]. According to simulation modeling conducted by Pedkenar et al. [15] there might be a more accurate estimate, but its implementation would be limited because of its advanced technical requirements.

Further, amongst the strengths of our study we also highlight the greater knowledge it provides on the characteristics of patients with multimorbidity admitted to a scarcely explored setting. Moreover, it empirically suggests for the first time the existence of an association between medication non-adherence and medication appropriateness.

The authors acknowledge the small sample size as the study main limitation. This is because sample size was estimated to test the primary hypothesis in a quasi-experimental (before–after) research. Nevertheless, this might have negatively affected statistical power, including the value barely below the level of significance for high PIP burden found in the multivariate analyses. Thereby, previous results warrant further investigation.

In addition, the use of the PIP burden as an estimate of medication appropriateness is not sufficiently standardized according to published literature. However, the association of cumulative PIP with poorer health outcomes in older people combined with the high prevalence of PIP in older patients with multimorbidity exposed to polypharmacy would support their use [42,48,49].

Additionally, medication adherence might be overestimated by using dispensing data from a six-month period due to situations such as drug oversupply or stockpiling [50]. Their influence on medication adherence estimates would be attenuated by the existence in the study setting of limitations for medication dispensing for periods longer than a month. Further, PDC accounts for overlapping days supplied to allow a more conservative estimate of adherence than MPR [14].

Finally, we cannot extend our results to every patient with multimorbidity because our findings are probably influenced by the high burden of PIP observed in study participants.

Despite its limitations, the present study provides new evidence regarding the need to implement patient-centered strategies aimed at improving effective prescribing in patients with multimorbidity to enhance both medication appropriateness and adherence. Such approaches should consider both objective and subjective medication adherence predictors as PIP burden and self-reported medication adherence, respectively. How these approaches would be feasibly implemented in clinical practice represents a future challenge. To date, strategies centered on enhancing effective prescribing have mainly been driven by pharmacists and focused on the relevance of educational aspects to optimize medication management and adherence [36]. Our results suggest effective prescribing should be more widely addressed by also considering interdisciplinary collaboration and medication appropriateness issues.

Further, ARMS has been proposed as a useful tool for identifying patients at risk of being non-adherents due to a suboptimal implementation of the dosing regimen and/or early discontinuation [13]. Future research might also explore how PIP burden influences medication adherence phases as per the ABC taxonomy [6].

## 5. Conclusions

The relationship between medication appropriateness and patient experience related to medication taking and refills measured through a self-report adherence method seems to play a key role in medication non-adherence in patients with multimorbidity and polypharmacy admitted to an intermediate care center. These findings provide a step forward in developing patient-centered strategies focus on improving effective prescribing in older patients with multimorbidity. These strategies should consider interdisciplinary collaboration and medication appropriateness issues.

## Figures and Tables

**Table 1:** Characteristics of non-institutionalized older patients with multimorbidity and polypharmacy admitted to an intermediate care center by medication adherence level.

Characteristic	Total N = 93	Non-Adherent ^1^ N = 74 (79.6%)	Adherent ^1^ N = 19 (20.4%)	*p* Value ^2^
**Demographic and Clinical Factors**				
Age in years (mean (SD))	83.0 (6.1)	83.0 (6.2)	83.1 (5.6)	0.926
Sex (*n* (%))				
*Male*	32 (34.4%)	27 (84.4%)	5 (15.6%)	0.405
*Female*	61 (65.6%)	47 (77.0%)	14 (23.0%)	
Chronic conditions (mean (SD))	7.40 (1.8)	7.57 (1.9)	6.74 (1.7)	0.079
Frailty (Frail-VIG) (mean (SD))	0.28 (0.11)	0.29 (0.11)	0.27 (0.11)	0.633
Activities of daily living (Barthel index) (mean (SD))	80.4 (19.7)	80.3 (20.0)	80.8 (19.2)	0.919
Cognitive impairment				
*Absence (MMSE > 24) (n (%))*	53 (57.0%)	43 (81.1%)	10 (18.9%)	
*Mild (MMSE 21–24) (n (%))*	23 (24.7%)	16 (69.6%)	7 (30.4%)	0.832
*Moderate (MMSE 10–20) (n (%))*	17 (18.3%)	15 (88.2%)	2 (11.8%)	
**Medication Factors**				
Long-term medications (mean (SD))	8.81 (2.8)	9.26 (2.8)	7.05 (2.2)	0.002
Hyperpolypharmacy (≥10 medications)	58 (62.4%)	42 (72.4%)	16 (27.6%)	
*No (n (%))*	35 (37.6%)	32 (91.4%)	3 (8.6%)	0.028
*Yes (n (%))*				
Medication regimen complexity score (MRCI) (mean (SD))	24.8 (10.7)	26.1 (10.7)	19.7 (9.7)	0.020
Medication regimen complexity (categorized)				0.022
*Low (MRCI < 20) (n (%))*	36 (38.7%)	23 (63.9%)	13 (36.1%)	
*Moderate-high (MRCI 20–39.5) (n (%))*	46 (49.5%)	42 (91.3%)	4 (8.7%)	
*Excessive (MRCI ≥ 40) (n (%))*	11 (11.8%)	9 (81.8%)	2 (18.2%)	
Anticholinergic and sedative risk score (DBI) (mean (SD))	0.99 (0.81)	1.03 (0.83)	0.82 (0.72)	0.311
Number of potentially inappropriate prescriptions (mean (SD))	2.55 (1.5)	2.69 (1.4)	2.00 (1.7)	0.074
Moderate (≥2) PIP burden				
*No (n (%))*	25 (26.9%)	17 (68.0%)	8 (32.0%)	0.093
*Yes (n (%))*	68 (73.1%)	57 (83.8%)	11 (16.2%)	
High (≥3) PIP burden				
*No (n (%))*	56 (60.2%)	40 (71.4%)	16 (28.6%)	0.017
*Yes (n (%))*	37 (39.8%)	34 (91.9%)	3 (8.1%)	
Self-reported adherence (ARMS-e total score) (mean (SD))	16.8 (4.1)	17.6 (4.1)	13.9 (2.9)	0.001
Self-reported adherence (categorized)				<0.001
*Optimal (ARMS-e = 12) (n (%))*	23 (24.7%)	11 (47.8%)	12 (52.2%)	
*Suboptimal (ARMS-e > 12) (n (%))*	70 (75.3%)	63 (90.0%)	7 (10.0%)	
Patient autonomy for medication administration at home				
*Independent (n (%))*	46 (49.5%)	36 (78.3%)	10 (21.7%)	0.564
*Partially assisted (n (%))*	32 (34.4%)	25 (78.1%)	7 (21.9%)	
*Totally assisted (n (%))*	15 (16.1%)	13 (86.7%)	2 (13.3%)	
Patient autonomy for medication refill at home				
*Independent (n (%))*	35 (37.6%)	26 (74.3%)	9 (25.7%)	0.093
*Partially assisted (n (%))*	13 (14.0%)	8 (61.5%)	5 (38.5%)	
*Totally assisted (n (%))*	45 (48.4%)	40 (88.9%)	5 (11.1%)	

^1^ Participants were divided by adherence level (considering dispensing date before admission) as non-adherents (multiple discretized proportion of days covered (PDC) < 80%; N = 74. 79.6%) or adherents (multiple discretized PDC ≥ 80%; N = 19. 20.4%). ^2^ Comparisons between adherent and nonadherent patients (by considering their multiple discretized PDC before admission) were performed using Student’s *t* test for parametric continuous variables or Mann–Whitney U test for nonparametric continuous variables. The chi-square test (or Fisher’s exact test where appropriate) was applied to compare both groups of categorical variables. Statistical significance was set at a two-sided *p* value of 0.05. Abbreviations: SD: standard deviation, MMSE: mini mental state examination, MRCI: Medication Regimen Complexity Index, DBI: Drug Burden Index, PIP: potentially inappropriate prescribing, ARMS-e: Spanish-version Adherence to Refills and Medications Scale.

**Table 2:** Logistic regression examining factors associated with medication non-adherence in older patients with multimorbidity and polypharmacy admitted to an intermediate care center.

Characteristic	Non-Adherence ^1^ (Bivariate Analysis ^2^)			Non-Adherence ^1^ (Multivariate Analysis ^2^)		
	OR	95% CI	*p* Value	OR	95% CI	*p* Value
Chronic conditions	1.30	0.97–1.74	0.083	-	-	-
Long-term medications	1.46	1.13–1.89	0.004	-	-	-
Hyperpolypharmacy (≥10 medications)						
*No*	1.00	Ref	Ref	-	-	-
*Yes*	4.06	1.09–15.15	0.037			
Medication regimen complexity score (MRCI)	1.07	1.01–1.14	0.025	-	-	-
Medication regimen complexity (categorized)						
*Low (MRCI < 20)*	1.00	Ref	Ref			
*Moderate-high (MRCI 20–39.5)*	5.94	1.73–20.32	0.005	-	-	-
*Excessive (MRCI* ≥ *40)*	2.54	0.48–13.60	0.275			
Number of potentially inappropriate prescriptions	1.47	0.96–2.24	0.079	-	-	-
Moderate (≥2) PIP burden						
*No*	1.00	Ref	Ref	-	-	-
*Yes*	2.44	0.85–7.04	0.099			
High (≥3) PIP burden						
*No*	1.00	Ref	Ref	1.00	Ref	Ref
*Yes*	4.53	1.22–16.89	0.024	3.90	0.95–15.99	0.059
Self-reported adherence (ARMS-e total score)	1.38	1.13–1.67	0.001	-	-	-
Self-reported adherence (categorized)						
*Optimal (ARMS-e = 12)*	1.00	Ref	Ref	1.00	Ref	Ref
*Suboptimal (ARMS-e > 12)*	9.82	3.17–30.42	<0.001	8.99	2.80–28.84	<0.001
Patient autonomy for medication refill at home						
*Independent*	1.00	Ref	Ref			
*Partially assisted*	2.77	0.83–9.19	0.096	-	-	-
*Totally assisted*	0.55	0.14–2.14	0.391			

^1^ Participants were divided by adherence level (considering dispensing date before admission) as non-adherents (multiple discretized proportion of days covered (PDC) < 80%; N = 74. 79.6%) or adherents (multiple discretized PDC ≥ 80%; N = 19. 20.4%). ^2^ Multivariable logistic regression analysis was conducted to assess the impact of each predictor on medication non-adherence before admission. The multivariate model was used for variables that had a *p* value < 0.10 in the bivariate analyses using stepwise regression. Statistical significance was set at a two-sided *p* value of 0.05. Abbreviations: OR: odds ratio, CI: confidence interval, MRCI: Medication Regimen Complexity Index, PIP: potentially inappropriate prescribing, ARMS-e: Spanish-version Adherence to Refills and Medications Scale.

## Data Availability

The datasets generated during and/or analyzed during the current study are available from the corresponding author on reasonable request.
